# Uncovering rate variation of lateral gene transfer during bacterial genome evolution

**DOI:** 10.1186/1471-2164-9-235

**Published:** 2008-05-20

**Authors:** Weilong Hao, G Brian Golding

**Affiliations:** 1 Department of Biology, McMaster University, Hamilton, Ontario L8S 4K1, Canada

## Abstract

**Background:**

Large scale genome arrangement, such as whole gene insertion/deletion, plays an important role in bacterial genome evolution. Various methods have been employed to study the dynamic process of gene insertions and deletions, such as parsimony methods and maximum likelihood methods. Previous maximum likelihood studies have assumed that the rate of gene insertions/deletions is constant over different genes. This assumption is unrealistic. For instance, it has been shown that informational genes are less likely to be laterally transferred than non-informational genes. However, how much of the variation in gene transfer rates is due to the difference between informational genes and non-informational genes is unclear. In this study, a Γ-distribution was incorporated in the likelihood estimation by considering rate variation for gene insertions/deletions between genes. This makes it possible to address whether a difference between informational genes and non-informational genes is the main contributor to rate variation of lateral gene transfers.

**Results:**

The results show that models incorporating rate variation fit the data better than do constant rate models in many phylogenetic groups. Even though informational genes are less likely to be laterally transferred than non-informational genes, the degree of rate variation for insertions/deletions did not change dramatically and remained high even when informational genes were excluded from the study. This suggests that the variation in rate of insertions/deletions is not due mainly to the simple difference between informational genes and non-informational genes. Among genes that are not classified as informational and among the informational genes themselves, there are still large differences in the rates that these genes are inserted and deleted.

**Conclusion:**

While the difference in informational gene rates contributes to rate variation, it is only a small fraction of the variation present; instead, a substantial amount of rate variation for insertions/deletions remains among both informational genes and among non-informational genes.

## Background

Gene insertions and deletions have been widely acknowledged to play an essential role in shaping bacterial genomes during evolution [1-4]. Parsimony methods have been employed to understand the process of gene insertions and deletions [5-8]. However, parsimony methods fail to distinguish parallel deletions and insertions on multiple branches [9-11]. The problem of parallel deletions and insertions can be overcome using maximum likelihood methods by making use of transition probabilities [12].

Recently a maximum likelihood method was employed to study gene insertions and deletions assuming constant rates across genes in a given genome [13]. However, the assumption of constant insertion/deletion rates among genes is unrealistic. For example, it has been shown that informational genes, such as those involved in transcription and translation, are less likely to be laterally transferred than are operational genes responsible for metabolic processes [14,15]. This observation forms the basis of the "complexity hypothesis". Unfortunately, causes of rate variation for insertions/deletions beyond the difference between informational genes and operational genes still remain unclear. A study of rate variation for gene insertions/deletions making use of the maximum likelihood method, therefore, becomes useful to address questions on rate variation for gene insertions/deletions.

**Figure 1:**
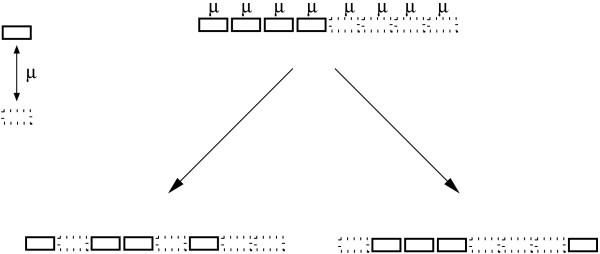
**The likelihood estimation is based on an assumption that each gene placeholder can change from gene presence to gene absence or vice versa with an ins/del rate *μ*.** The rate *μ *might vary among genes.

Here, a Γ-distribution has been incorporated into a maximum likelihood estimation of gene insertion/deletion rates (Figure 1). After incorporating rate variation for gene insertions/deletions among genes, the likelihood was improved significantly over a constant rate model using the same set of data from the *Bacillus *group as in [13]. The method was applied to 173 complete bacterial genomes in 25 phylogenetic groups; 20 groups showed significantly better fits to the data with rate variation for gene insertions/deletions. The remaining five groups did not show significantly better fits to the data with rate variation. Furthermore, the removal of informational genes from the likelihood estimation contributes little change in terms of the rate variation parameter *α *for gene insertions/deletions. This is the case despite informational genes having significantly lower rates of insertions/deletions than non-informational genes.

The results reveal that rate variation of gene insertions/deletions is much more complex than simply a difference between informational genes and operational genes; instead, a high degree of rate variation for insertions/deletions remains among both informational genes and among non-informational genes.

## Results

**Figure 2:**
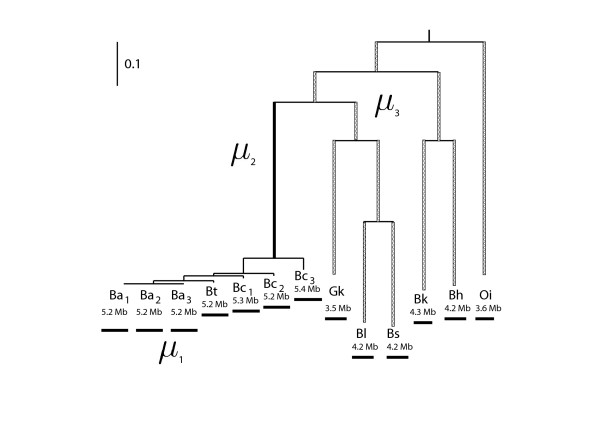
**Different insertion/deletion rates were assumed on the phylogeny.** Case 1: a single constant rate throughout the phylogeny (*μ*_1 _= *μ*_2 _= *μ*_3_). Case 2: two rates differentiate the Bc group (*μ*_1_, *μ*_2 _= *μ*_3_). Case 3: three rates differentiate the Bc group and the branch leading to this group (*μ*_1_, *μ*_2_, *μ*_3_).

The same set of data from the *Bacillus *group in [13] was used to initially test the performance of the models incorporating rate variation. Following the previous study, three rate-conditions (*μ*_1 _= *μ*_2 _= *μ*_3_; *μ*_1_, *μ*_2 _= *μ*_3_; *μ*_1_, *μ*_2_, *μ*_3_;) were assumed (see Figure 2), each one was further extended by adding rate variation.

The likelihood of the models was improved significantly by incorporating a Γ-distribution in rate variation models compared with relevant constant rate models (*χ*^2 ^= 2ΔLn*L *≫ 3.84 with d.f. = 1, see Table 1). Of the three models, the likelihood of the single-constant rate model was improved the most by incorporating rate variation. In the single rate model with a Γ-distribution, the MLE (maximum likelihood estimation) rate of insertions/deletions is 5.29, which is much greater than the rate 0.51 of the constant rate model. There is a high degree of rate variation for gene insertions/deletions, since the rate variation parameter *α *is 0.37; indicating that there is a small subset of genes with rapid gene turn-over.

**Table 1:** Incorporating rate variation in the maximum likelihood estimation using the same set of data in Hao and Golding (2006).

Rate	Gamma Distribution			Constant Rate		ΔLn*L*
			
	*α *value	MLE	Ln*L*	MLE	Ln*L*	
*μ*_1 _= *μ*_2 _= *μ*_3_	0.37	5.29	-39765	0.51	-40277	512

*μ*_1 _	5.83	4.73	-36874	4.42	-36902	28
*μ*_2 _= *μ*_3_		0.37		0.35		

*μ*_1_	3.04	4.49	-36042	3.92	-36128	86
*μ*_2_		0.32		0.28		
*μ*_3_		1.67		1.23		

The two-rate and three-rate models both assumed different rates on certain parts of the phylogeny. After incorporating rate variation, they both showed significantly better fits to the data than did the single rate model (Table 1), but the MLE rates are similar to those estimated from the constant rate models. In the two-rate model, the rates with rate variation in a Γ-distribution are 4.73, 0.37 versus 4.42, 0.35 with constant rates. Similarly, in the three-rate model, the rates with rate variation are 4.49, 0.32, 1.67 versus 3.92, 0.28, 1.23 with constant rates. Both models consistently support that recently transferred genes tend to have high rates of gene insertions/deletions as noted in [13]. Both cases showed lower levels of rate variation (greater *α *values) compared with the single-rate model (5.83 and 3.04 versus 0.37 of *α *values), even though, incorporating rate variation also improved the likelihood significantly.

**Figure 3:**
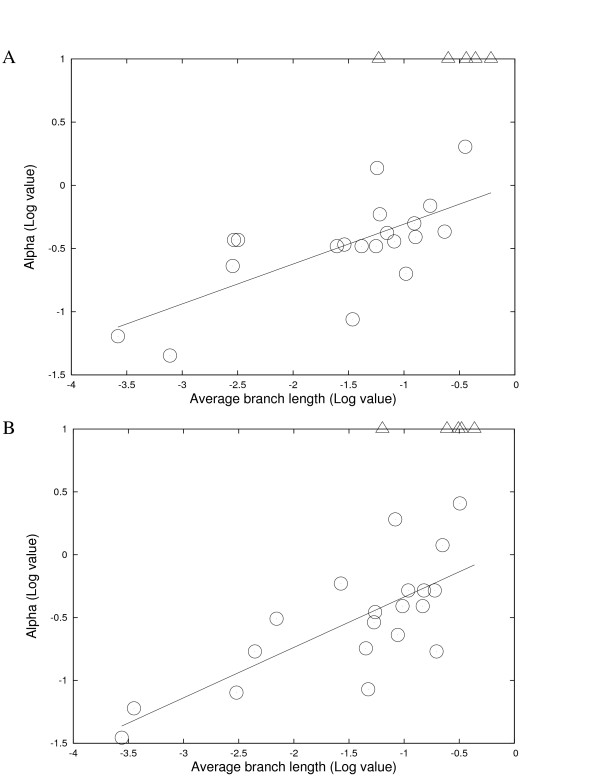
**Average branch length and optimized *α *in a Γ distribution in each different group.** The five groups with an infinite *α *value are shown in open triangles. They were not used for estimating the regression lines. A, Estimation was based on the select-genes trees, *y *= 0.316*x *+ 0.009 (*R*^2 ^= 0.480, *P *≃ 0.0007); B, Estimation was based on the common-genes trees, *y *= 0.401*x *+ 0.064 (*R*^2 ^= 0.562, *P *≃ 0.0001).

To gain a clearer picture of rate variation of lateral gene transfer in the domain of bacteria, the study was expanded to 173 complete bacterial genomes in 25 phylogenetic groups (Tables 2 and 3). For each phylogenetic group, two phylogenies were constructed. One (the select-genes tree) is based on a group of selected genes, the other (the common-genes tree) is based on genes present among the relevant taxa as described in the Methods section. Of the 25 phylogenetic groups, 20 groups showed a fairly high level of rate variation for gene insertions/deletions among genes, and the confidence interval falls into a small range for each *α *value (Table 4). It is also striking that estimates using phylogenies on different set of genes or using different phylogenies are similar (Tables 2, 3, and 4). Five groups did not show a significant level of rate variation for gene insertions/deletions (*α *is ∞). The five groups are *Candidatus*, *Ehrlichia*, *Lactobacillus*, *Mycoplasma*, and *Synechococcus*. The lower boundary of each infinite *α *value was also estimated. The *Ehrlichia *group shows a very broad interval range for the "maximum" likelihood value and the lower boundary of *α *value is 0.39 (Table 4). The undistinguished difference between the rate variation model and the constant rate model in *Ehrlichia *might be due to the limited size of data (gene families) and/or accelerated evolution at the sequence level in this intracellular group. The relationship between the rate variation parameter *α *in a Γ distribution and the average branch length of each group was examined. Figure 3 shows that there is a positive correlation between the rate variation parameter *α *and the average branch length of each group. Closely related groups tend to have a higher degree of rate variation for gene insertions/deletions among genes, while distantly related groups tend to have a lower level of rate variation for gene insertions/deletions. This suggests that the observation of rate variation is a strong local phenomenon and becomes blurry over evolutionary time.

**Table 2:** Insertion/deletion rates among different phylogenetic groups estimated in rate variation model. Estimation was based on the select-genes trees.

Group	Taxa	Genome Size	Branch Length	Rate Variation			Constant Rate		ΔLn*L*
			
				*α *value	MLE	Ln*L*	MLE	Ln*L*	
*Bacillus*	13	4.9	0.126886	0.39	4.79	-40187	0.51	-40545	358*
*Brucella*	4	2.1	0.000775	0.053	126.82	-1214	22.77	-1253	39*
*Burkholderia*	7	3.8	0.028971	0.35	25.58	-17258	3.54	-17717	459*
*Candidatus*	4	1.3	0.608223	∞	0.36	-4635	0.36	-4635	0
*Chlamydophila*	7	1.2	0.081640	0.41	0.36	-1427	0.22	-1451	25*
*Clostridium*	5	3.3	0.123806	0.50	11.93	-9544	9.84	-9769	225*
*Corynebacterium*	5	3.0	0.172207	0.79	2.48	-8537	0.71	-8600	63*
*Ehrlichia*	5	1.5	0.059080	∞	0.27	-832	0.27	-832	0
*Escherichia*	7	5.0	0.002852	0.23	51.34	-10689	12.53	-11236	547*
*Helicobacter*	5	1.7	0.232504	0.47	1.75	-3966	1.16	-4133	167*
*Lactobacillus*	6	2.2	0.250368	∞	0.44	-9302	0.44	-9302	0
*Mycobacterium*	6	4.2	0.070373	0.41	19.94	-14693	1.63	-14718	25*
*Mycoplasma*	12	0.9	0.441286	∞	0.13	-8448	0.13	-8448	0
*Prochlorococcus*	5	1.9	0.356990	2.42	0.17	-4414	0.15	-4421	7*
*Pseudomonas*	8	6.3	0.057175	1.66	2.09	-27117	1.32	-27231	114*
*Rhodopseudomonas*	4	5.3	0.060450	0.59	2.73	-9342	1.43	-9433	91*
*Rickettsia*	5	1.3	0.055962	0.33	3.70	-3172	1.30	-3302	130*
*Salmonella*	5	4.8	0.002930	0.37	12.19	-5283	7.97	-5356	73*
*Shigella*	6	4.6	0.003188	0.37	39.69	-9753	16.25	-10064	311*
*Staphylococcus*	13	2.8	0.034391	0.090	407.56	-12283	19.77	-14514	2231*
*Streptococcus*	19	2.0	0.041459	0.34	12.76	-24015	9.39	-26146	2131*
*Synechococcus*	5	2.6	0.364362	∞	0.31	-7320	0.31	-7320	0
*Vibrio*	5	3.2	0.104170	0.21	11.72	-8623	0.74	-8883	260*
*Xanthomonas*	6	5.1	0.024790	0.33	18.00	-9388	3.21	-9714	326*
*Yersinia*	6	4.6	0.000263	0.064	261.75	-3236	46.77	-3606	370*

*Significant improvement

**Table 3:** Insertion/deletion rates among different phylogenetic groups estimated in rate variation model. Estimation was based on the common-genes trees.

Group	Taxa	Genome Size	Branch Length	Rate Variation			Constant Rate		ΔLn*L*
			
				*α *value	MLE	Ln*L*	MLE	Ln*L*	
*Bacillus*	13	4.9	0.147413	0.39	3.89	-40017	0.44	-40278	261*
*Brucella*	4	2.1	0.000275	0.035	382.06	-1264	44.50	-1387	123*
*Burkholderia*	7	3.8	0.044985	0.18	181.93	-17467	2.14	-18391	924*
*Candidatus*	4	1.3	0.433912	∞	0.49	-4722	0.49	-4722	0
*Chlamydophila*	7	1.2	0.096709	0.39	0.30	-1439	0.19	-1466	27*
*Clostridium*	5	3.3	0.151053	0.52	8.49	-9529	7.23	-9756	227*
*Corynebacterium*	5	3.0	0.223104	1.19	2.48	-8492	0.49	-8509	17*
*Ehrlichia*	5	1.5	0.063546	∞	0.24	-831	0.24	-831	0
*Escherichia*	7	5.0	0.006966	0.31	13.43	-10654	4.82	-11006	352*
*Helicobacter*	5	1.7	0.189516	0.52	1.75	-3961	1.05	-4109	148*
*Lactobacillus*	6	2.2	0.331530	∞	0.33	-9241	0.33	-9241	0
*Mycobacterium*	6	4.2	0.087643	0.23	191.03	-14660	1.63	-14718	25*
*Mycoplasma*	12	0.9	0.244815	∞	0.22	-8214	0.22	-8214	0
*Prochlorococcus*	5	1.9	0.319829	2.56	0.19	-4413	0.17	-4419	6*
*Pseudomonas*	8	6.3	0.083078	1.91	1.06	-26994	0.91	-27130	136*
*Rhodopseudomonas*	4	5.3	0.109002	0.52	1.75	-9352	0.77	-9459	107*
*Rickettsia*	5	1.3	0.054524	0.35	3.49	-3181	1.36	-3310	129*
*Salmonella*	5	4.8	0.003016	0.080	96.48	-5214	7.97	-5351	137*
*Shigella*	6	4.6	0.004433	0.17	131.93	-9784	11.39	-10327	543*
*Staphylococcus*	13	2.8	0.047252	0.085	279.41	-11390	14.76	-14581	3191*
*Streptococcus*	19	2.0	0.053313	0.29	18.71	-24327	13.19	-26553	2226*
*Synechococcus*	5	2.6	0.310236	∞	0.33	-7531	0.33	-7531	0
*Vibrio*	5	3.2	0.196671	0.17	10.02	-8642	0.37	-8939	260*
*Xanthomonas*	6	5.1	0.026682	0.59	6.33	-9385	2.73	-9541	156*
*Yersinia*	6	4.6	0.000355	0.060	221.14	-3172	31.15	-3467	295*

*Significant improvement

**Table 4:** The confidence interval of the *α *value in a Γ distribution of each group. Estimates based on the select-genes tree and the common-genes tree are shown.

Group	Taxa	Lower Boundary*		*α *value		Upper Boundary*	
			
		Select	Common	Select	Common	Select	Common
*Bacillus*	13	0.38	0.38	0.39	0.39	0.40	0.40
*Brucella*	4	0.029	0.026	0.053	0.035	0.066	0.042
*Burkholderia*	7	0.32	0.17	0.35	0.18	0.37	0.19
*Candidatus*	4	108	108	∞	∞	-	-
*Chlamydophila*	7	0.28	0.28	0.41	0.39	0.57	0.59
*Clostridium*	5	0.44	0.47	0.50	0.52	0.56	0.58
*Corynebacterium*	5	0.56	0.84	0.79	1.19	1.03	1.71
*Ehrlichia*	5	0.39	0.39	∞	∞	-	-
*Escherichia*	7	0.20	0.26	0.23	0.31	0.27	0.35
*Helicobacter*	5	0.39	0.45	0.47	0.52	0.56	0.63
*Lactobacillus*	6	11	12	∞	∞	-	-
*Mycobacterium*	6	0.38	0.22	0.41	0.23	0.49	0.25
*Mycoplasma*	12	67	72	∞	∞	-	-
*Prochlorococcus*	5	1.40	1.51	2.42	2.56	4.45	4.45
*Pseudomonas*	8	1.22	1.52	1.66	1.91	1.62	2.14
*Rhodopseudomonas*	4	0.49	0.44	0.59	0.52	0.75	0.66
*Rickettsia*	5	0.27	0.28	0.33	0.35	0.42	0.41
*Salmonella*	5	0.30	0.071	0.37	0.080	0.46	0.10
*Shigella*	6	0.31	0.15	0.37	0.17	0.41	0.19
*Staphylococcus*	13	0.081	0.080	0.090	0.085	0.099	0.090
*Streptococcus*	19	0.31	0.28	0.34	0.29	0.35	0.32
*Synechococcus*	5	108	108	∞	∞	-	-
*Vibrio*	5	0.19	0.16	0.21	0.17	0.24	0.19
*Xanthomonas*	6	0.29	0.52	0.33	0.59	0.37	0.70
*Yersinia*	6	0.054	0.053	0.064	0.060	0.077	0.071

*at 5% level, and the lower boundary of any ∞ *α *value is shown in integers except in the *Ehrlichia *group, which has a lower boundary 0.39.

**Figure 4:**
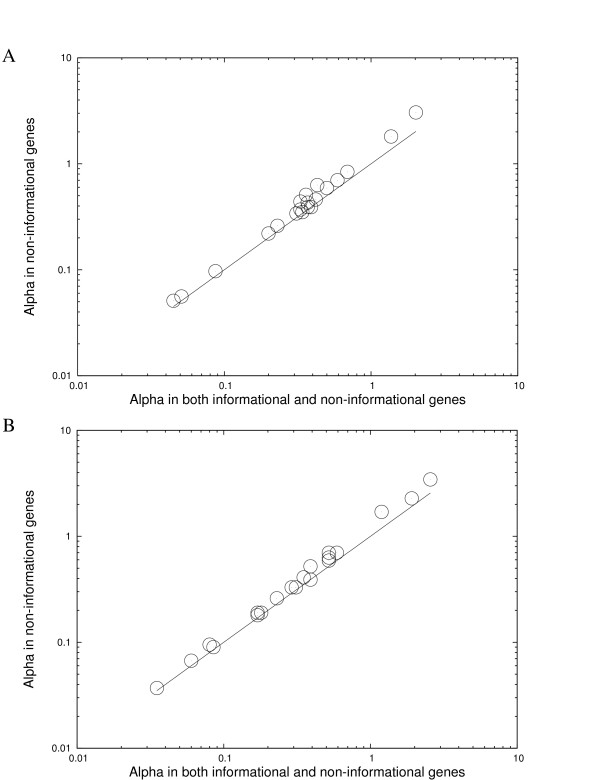
**No dramatic change in *α *values in a Γ-distribution when informational genes were excluded from the estimation.** A, Estimation was based on the select-genes trees; B, Estimation was based on the common-genes trees. The *y *= *x *line is also shown.

MLE estimates of gene insertions/deletions rates in informational genes and in non-informational genes were estimated separately in the absence of rate variation. The rates in informational genes are lower than those in non-informational genes in all groups, but none of them are zero (Additional file 1). This is consistent with previous studies that have shown informational genes have slower rates of gene insertions/deletions than non-informational genes [14,15], but they are not completely free of gene movement [16]. To evaluate whether the difference in informational genes contributes the most to rate variation of gene transfers, maximum likelihood estimation with rate variation for gene insertions/deletions was conducted by excluding all informational genes. The *α *values excluding informational genes are remarkably similar to those including informational genes (Figure 4), even though the *α *values are slightly increased when excluding informational genes. However this could be because informational genes tend to be more conserved. We therefore, excluded the same number of most conserved genes (e.g. genes present in all taxa in each group). In this case, a greater increase in *α *values was observed than that observed when informational genes were excluded (Table 5). The ratio of the increase of *α *value after excluding informational genes over the increase in *α *value after excluding the same number of most conserved genes was calculated (Table 5). If all informational genes are as rarely transferred as are the most conserved genes, one should expect that the ratio is close to 1. However, the ratio in most groups (14 of 20 groups in each set of analysis) is smaller than 0.5, suggesting that the effect of excluding informational genes is similar to that of excluding random genes rather than that of excluding the most conserved genes. Rate variation for gene insertions/deletions in non-informational genes still remained high after the genes that do not have significant matches to any genes in COG classification were removed (Additional file 2). Furthermore, the level of rate variation in informational genes is significant in most groups (Additional file 3). Thus, rate variation for gene insertions/deletion is not mainly due to the difference between informational genes and non-informational genes, but instead, a substantial amount of variation for gene insertions/deletions is observed in both informational and non-informational genes. In other words, the "complexity hypothesis" only explains a small part of the variation in rate of gene insertions/deletions.

**Table 5:** Different *α *values in a Γ distribution after excluding certain genes. Estimates based on the select-genes tree and the common-genes tree are shown.

Group	Original				Genes removed				Difference^*a *^	
		
			Random		Informational		Conserved		(Ratio)	
					
	Select	Common	Select	Common	Select	Common	Select	Common	Select	Common
*Bacillus*	0.39	0.39	0.38	0.39	0.39	0.39	0.47	0.47	0.11	0.00
*Brucella*	0.053	0.035	0.052	0.035	0.057	0.037	0.072	0.044	0.25	0.22
*Burkholderia*	0.35	0.18	0.35	0.16	0.37	0.19	0.50	0.21	0.13	0.60
*Candidatus*	∞	∞	∞	∞	∞	∞	∞	∞	-	-
*Chlamydophila*	0.41	0.39	0.40	0.41	0.54	0.52	0.56	0.52	0.87	1.00
*Clostridium*	0.50	0.52	0.49	0.52	0.59	0.63	1.06	1.12	0.18	0.18
*Corynebacterium*	0.79	1.19	0.80	1.19	1.01	1.70	1.75	3.06	0.22	0.27
*Ehrlichia*	∞	∞	∞	∞	∞	∞	∞	∞	-	-
*Escherichia*	0.23	0.31	0.25	0.31	0.26	0.33	0.31	0.39	0.17	0.25
*Helicobacter*	0.47	0.52	0.47	0.52	0.59	0.70	0.66	0.79	0.63	0.67
*Lactobacillus*	∞	∞	∞	∞	∞	∞	∞	∞	-	-
*Mycobacterium*	0.41	0.23	0.40	0.24	0.49	0.26	0.65	0.35	0.36	0.18
*Mycoplasma*	∞	∞	∞	∞	∞	∞	∞	∞	-	-
*Prochlorococcus*	2.42	2.56	2.46	2.56	3.05	3.44	5.50	6.95	0.19	0.20
*Pseudomonas*	1.66	1.91	1.64	1.91	2.16	2.28	3.80	3.86	0.24	0.19
*Rhodopseudomonas*	0.59	0.52	0.59	0.56	0.70	0.59	0.84	0.66	0.44	0.30
*Rickettsia*	0.33	0.35	0.33	0.37	0.44	0.41	0.53	0.50	0.55	0.31
*Salmonella*	0.37	0.080	0.39	0.080	0.44	0.095	0.49	0.102	0.50	0.68
*Shigella*	0.37	0.17	0.35	0.17	0.39	0.18	0.50	0.21	0.27	0.25
*Staphylococcus*	0.090	0.085	0.093	0.085	0.097	0.090	0.116	0.114	0.17	0.17
*Streptococcus*	0.34	0.29	0.34	0.29	0.37	0.33	0.47	0.41	0.23	0.33
*Synechococcus*	∞	∞	∞	∞	∞	∞	∞	∞	-	-
*Vibrio*	0.21	0.17	0.22	0.18	0.23	0.19	0.27	0.23	0.20	0.20
*Xanthomonas*	0.33	0.59	0.33	0.59	0.37	0.70	0.39	0.74	0.67	0.73
*Yersinia*	0.064	0.060	0.064	0.063	0.070	0.067	0.075	0.071	0.55	0.50

^*a *^The difference was calculated from $\frac{\alpha_{\text{informational}}-\alpha_{\text{random}}}{\alpha_{\text{conserved}}-\alpha_{\text{random}}}$

## Discussion

The accuracy of maximum likelihood estimation of gene indel rates is dependent on the presence of a robust phylogeny for the genomes under study. Phylogenies obtained from single genes can sometimes be distorted due to rampant LGT [2,6,17,18] and rRNA sequences may not be useful due to the lack of informative characters differentiating closely related species and varying functional constraints over the molecule [19,20]. We used a concatenated DNA sequence obtained by joining the gene sequences that are commonly present in many bacterial genomes. For the select-genes tree construction, a set of genes were chosen from those reported in previous studies [21,22]. If there is more than one phylogeny generated for a group, all phylogenies were used and weighted by their occurrence to overcome the uncertainty of using just one. To avoid the confounding effects of duplication during evolution [23,24], duplicated genes were removed from phylogeny construction. Due to the broad spectrum of species analyzed in this study, there are few genes free of both duplication and lateral gene transfer across all groups. Consequently, the genes used for phylogeny reconstruction may be different between groups (details are given as supplementary information at [25]). To assess the robustness of each select-genes tree, the common-genes trees were reconstructed using genes present in all members of each phylogenetic group. When the common-genes tree and the select-genes tree are not topologically identical, a supertree was constructed. There are 12 groups that have an identical topology between the select-genes tree and the common-genes tree. The remaining 13 groups do not show an identical topology between the select-genes tree and the common-genes tree. Please note that many differences are due to either the lack of phylogenetic signal at the tips of a phylogeny or the placement of the root (see supplementary information [25] for more details).

One way to achieve a more accurate phylogeny is to make use of a large number of genes in comprehensive phylogenetic studies, such as supermatrics (concatenated genes) and supertrees [26-30]. The more data included in a phylogenomic analysis, the more likely to overcome possible stochastic errors [27,31]. In this study, the common-genes tree was not always favored by the supertree over the select-genes trees. Indeed, there are two groups that the select-genes tree is supported by the supertree (Additional file 4). Slow evolving genes are sometimes more informative for phylogeny construction, since fast evolving genes might cause problems such as long branch attraction [28,32]. The tree length of each gene was computed from each phylogeny and plotted in (Additional file 5). It is clear that the selected genes for phylogeny construction have relatively slow evolutionary rates compared with all common genes. There are 4 groups whose supertree does not support the select-genes tree or the common-genes tree. The lack of congruence in these groups is likely due to insufficient taxon sampling. [27]. More accurate trees might be obtained as more complete genome sequences become available. Importantly, the maximum likelihood estimates based on the common-genes trees are remarkably similar with the estimates based on the select-genes trees (Tables 2, 3, 4, 5 and Figures 3, 4). For the 4 groups that do not have an identical topology between the select-genes tree and the common-genes tree, the results reported are based on the supertree topology (branch lengths were estimated from the selected genes). The results are again similar to those based on the select gene tree and those based on the common-genes tree (data not shown).

To further explore how much, if at all, different phylogenies might alter the results, maximum likelihood estimation based on possible alternative topologies was investigated. One hundred bootstraps from the alignment of the common genes were generated for each group. For the select-genes, possible alternative topologies were obtained from the MRBAYES output. If there are more than 10 distinct topologies, the top 10 ones according to their likelihood were chosen for further maximum likelihood estimation. The maximum likelihood estimates are shown in Additional files 6 and 7. It is clear that the *α *values are similar among different phylogenies. The removal of informational genes results in little change on the rate variation parameter *α*, and this holds true for each phylogeny. Furthermore, the likelihood estimations in the *Bacillus *group based on a phylogeny constructed from different genes (Tables 2 and 3) are similar with those based on the phylogeny of the previous study (Tables 1). The slight difference is due to the removal of short sequences in this study and differences in the phylogenies constructed from different sequences. The results do not, therefore, seem to be an artifact of the genes included or the phylogeny reconstruction. Informational genes are known to be less likely to undergo lateral gene transfer [14], which is also the core of the "complexity hypothesis" [15,33]. In this study, informational genes were found to have lower rates of gene insertions/deletions compared with non-informational genes (Additional file 1). However, no group has an insertion/deletion rate equal to 0, suggesting that informational genes are not completely free of gene movement. In fact, several ribosomal protein coding genes are deleted from *Streptococcus mutans *(Additional file 8). The rate variation parameter, *α*, change after excluding informational genes is similar to the *α *change after randomly removing genes rather than the *α *change after excluding the most conserved genes (Additional file 9). Furthermore, different cutoffs used in identifying informational genes only resulted in variation of the number of informational genes but did not affect the degree of rate variation for insertions/deletions in non-informational genes (Additional file 10). There is a great deal of rate variation for gene insertions/deletions in non-informational genes and also there is a significant level of rate variation in informational genes. In other words, the different rates between informational genes and non-informational genes as shown in the "complexity hypothesis" can only explain a small part of rate variation for gene insertions/deletions. Similarly, our simulation study showed that the high level of rate variation can not be explained solely by the fast turn-over rates of recently transferred genes (Additional file 11).

It has been suggested that different cutoff thresholds for identifying homologues might affect the identification of some gene gains [34], but different thresholds result in little change on the number of gene families [35] and the rates of gene insertions/deletions [36]. In this study, results using different thresholds were similar (data not shown). It is important to note that gene duplication was not taken into consideration in this study, since our focus was on insertions/deletions of gene families rather than intraspecific gene family duplication. This avoids the difficulty of distinguishing some gene transfer from gene duplication [4,11]. Recently, some studies have suggested that duplicated genes or genes that have a high duplicability propensity might be more likely to be involved in lateral gene transfer [37,38]. Methods incorporating gene duplication information are desirable for future studies.

For the 20 groups that showed a significant improvement in likelihood by adding rate variation, there is a positive association between the rate variation parameter *α *and the average branch length. Higher degrees of rate variation for gene insertions/deletion are expected to be observed in closely related groups. The seven closely related *Bacillus *genomes in the Bc group were analyzed separately and, as expected, a high degree of rate variation was observed (data not shown). Similarly, the five groups that have an infinite *α *value show fairly high levels of divergence within the group in terms of the average branch length. An acceleration of sequence evolution in the endosymbiont genomes has been acknowledged [39]. The accelerated rates of evolution might affect the branch lengths of the phylogeny used in the analyses and might also affect the identification of homologues within each phylogenetic group. Four endosymbiont groups showed strong accelerated rates of evolution. They are *Candidatus*, *Ehrichia*, *Mycoplasma*, and *Richettsia*. Analyses after the removal of these four groups also showed similar results (data not shown).

There are two possible explanations for the correlation between the average branch length and the *α *value. First, it is possible that the observed correlation is due to the lack of power of maximum likelihood estimation in distantly related groups. Previously, it has been shown that comparison among distantly related species tends to infer lower rates of insertions/deletions [13]. On the other hand, if maximum likelihood estimation in the study has enough power in distantly related groups, the results might suggest that rate variation for insertions/deletions has a strong local effect and becomes weaker as evolution in progress. This strong local effect might be, at least partially, due to a high variability in recently transferred genes. It is known that many of recently transferred genes are under faster rates of evolution and might be eliminated from the genome rapidly [8,13], and while some transferred genes that play roles in long term adaptation might become fixed [40-43] and integrated into the functional network [44,45]. Maximum likelihood estimations from simulated data showed support for these explanations. When the number of insertions/deletions increased, a larger proportion of insertions/deletions became undetectable, at the same time, sister taxa shared less common genes and have more unique genes (Additional file 11). If this correlation holds true, after a long enough time period, one should expect rate variation becomes undetectable. Hence, over a long time period, genes would have roughly the same chance to be transferred. This has been shown in some recent studies. By examining the *Cyanobacteria *group, Zhaxybayeva *et al. *reported that genes from all functional categories are subject to gene transfer [46]. In addition, it was suggested that among all sequenced gene families, at least two-thirds and probably all, have been affected by LGT at some time in their evolutionary past [35].

On the other hand, not many genes in a genome are shown to be affected by lateral gene transfer when comparing closely related species; This might be partially able to explain the contradictory views of lateral gene transfer at different phylogenetic scales. It has been reported that the genes from closely related species tend to have clearer tree-like relationship than the ones from distantly related species [47,48] and the studies analyzing genomes in different degrees of divergence do not show congruent results [18,49]. There may be several sources of noise in the data in Figure 3. It is plausible that different phylogenetic groups might have slightly different mechanisms of preventing lateral gene transfer or selectively retaining certain foreign genes. In fact, it is known that bacteria are able to selectively retain foreign genes with certain sequence features, such as codon usage [50] and GC content [51,52]. The possibility of lateral gene transfers into a genome could also be affected by other internal or external environmental factors, such as genome size, carbon utilization, isolated niches, and biochemical properties [53-55]. However, there is no evidence found in this study that the degree of rate variation for gene insertions/deletions across genes is associated with genome size (data not shown).

## Conclusion

Maximum likelihood models incorporating rate variation allow us to evaluate the contribution to rate variation of gene insertions/deletions between informational genes and non-informational genes. Consistent with the "complexity hypothesis", informational genes are less likely to be laterally transferred than non-informational genes. However, the difference between informational genes and non-informational genes is only a small fraction of the variation present; instead, a substantial amount of rate variation for insertions/deletions remains among both informational genes and among non-informational genes. Furthermore, the observation of rate variation has a strong local effect and becomes blurry over evolutionary time.

## Methods

A maximum likelihood model was used as described in [13]. In brief, gene presence or gene absence was treated as a binary character (0,1) state (Figure 1). Given the evolutionary history, the probability of gene movement can be computed from insertion and deletion rates. Like the maximum likelihood estimation of a phylogeny using DNA sequence, the likelihood of a character state at any node on a given phylogeny can be calculated from the character states in the immediate descendant nodes. The likelihood of the gene phyletic pattern *i *(gene family *i*) at the last common ancestral node is *L*_*i*_. In the maximum likelihood estimation, the rates were optimized to find those rates that maximized the likelihood of all gene patterns. Rate variation for gene insertions/deletions among genes was taken into account in the model in a similar manner as nucleotide rate heterogeneity in phylogeny reconstruction [56,57]. A discrete Γ model with nine rate categories (*M *= 9 categories) was implemented in the maximum likelihood estimation. Thus if the likelihood on gene family *i *with rate *μ *is *L*^*i*^(*μ*), and the density function of the distribution of rates is *f*(*μ*), the likelihood on site *i *will be

$$L^{i}=\int_{0}^{\infty }f(\mu )L^{i}(\mu )d\mu =\sum_{j=1}^{M}p_{j}L^{i}(\mu_{j}).$$

Since the genes absent in all of the taxa are unobservable, the results must be corrected for missing data. Hao and Golding (2006) used the correction for missing data as was used for missing restriction sites in [58], and the results are then made conditional on observing the gene present in at least one species. This is

$$L_{+}=\frac{L}{1-L_{-}}.$$

Here *L*_- _is the likelihood of a gene being absent in all taxa while *L*_+ _is the likelihood of the gene present in at least one genome from the observed data. After incorporating a discrete Γ model, the likelihood of observing the pattern of gene family *i *will be

$$L_{+}^{i}=\sum_{j=1}^{M}p_{j}\frac{L^{i}(\mu_{j})}{1-L_{-}^{i}(\mu_{j})}.$$

At the root of the tree we can compute the overall likelihood as

$$Q_{+}=\prod_{i=1}^{N}L_{+}^{i}=\prod_{i=1}^{N}\sum_{j=1}^{M}p_{j}\frac{L^{i}(\mu_{j})}{1-L_{-}^{i}(\mu_{j})}.$$

Here *N *is the total number of gene families. To estimate the maximum likelihood, the ins/del rates together with the rate variation parameter *α *in a Γ distribution were optimized to find those rates/values that maximized the likelihood of observing the gene patterns.

The same set of data from the *Bacillus *group in [13] were used to test the performance of the likelihood model with rate variation. As was done in the previous study, three models were examined (Figure 2) in an attempt to capture some of the major differences among the genomes. In brief, the rate on the branches of the Bc group (including Ba, Bc, and Bt) is *μ*_1_, the rate on the branch leading to the Bc group is distinguished as *μ*_2_, the rate on the remaining branches is *μ*_3_. Three models were examined; one with all branches evolving at the same rate (*μ*_1 _= *μ*_2 _= *μ*_3_), one with just the Bc group evolving at a separate rate (*μ*_1_, *μ*_2 _= *μ*_3_) and one with each of the three groups of branches evolving at separate rates (*μ*_1_, *μ*_2_, *μ*_3_). The likelihood of each model incorporating rate variation was compared with that of the relevant constant rate model. In each case, the likelihood of the constant rate model was improved significantly by incorporating a Γ-distribution for insertion/deletion rates (Table 1).

To apply the improved maximum likelihood estimation to a broad spectrum in the bacterial domain, 173 complete bacterial genomes in 25 phylogenetic groups (including *Bacillus*) were examined. Genomes were selected to be within the same group based on the same genus name in the NCBI taxonomy database and whenever at least four genomes from the same genus were completely sequenced. Following previous studies, *Oceanobacillus iheyensis *and *Geobacillus kaustophilus *were included in the *Bacillus *group [13] and *Ureaplasma urealyticum *was included in the *Mycoplasma *group [8]. Since some highly diverged *Synechococcus *species are closely related to *Prochlorococcus *species [59], the group of *Synechococcus *in this study only includes *Synechococcus *sp. strains. Genome sequences were obtained from the NCBI database [60]. Sixteen non-ribosomal protein coding genes from commonly present genes [21,22] were chosen for phylogeny construction, and they are *argS*, *gcp*, *gltX*, *hisS*, *infB*, *ksgA*, *lysS*, *metG*, *nusA*, *nusG*, *pheS*, *proS*, *rpoA*, *secY*, *serS*, and *ychF*. In each group, any duplicated genes of these 16 genes were excluded from phylogeny construction for that group. The phylogeny of each group was constructed from the concatenated DNA sequences of these genes using MRBAYES [61] (200,000 generations sampled every 100 generations with a Γ distribution model and invariant class). For convenience, this tree is called the select-genes tree. The species information of each group together with outgroup information, genes used for phylogeny construction of each group, and the best supported phylogenetic tree are given as supplementary information at [25]. If more than one possible phylogeny was generated for a group, all possible phylogenies were used for further analysis, weighed by their posterior probabilities.

The robustness of these phylogenies was further assessed by concatenating all common genes from each group (labelled the common-genes tree to distinguish it from the select-genes tree). As for selected genes, common genes that have paralogs were excluded from the analysis, to avoid the confounding effects of duplication. The number of genes (and characters) from each group are given in Additional file 4. Sequence alignment was performed individually for each gene using MUSCLE [62]. Aligned sequences were concatenated for phylogenetic analysis. Since MRBAYES [61] has a limitation for the maximum number of characters, DNAML in the PHYLIP package was used instead and the rate variation parameter alpha was estimated using the PUZZLE program [63].

A supertree method was then employed for the groups in which the select-genes tree and common-genes tree are not topologically identical. Genes present in at least 4 taxa were used for phylogeny construction. A supertree was computed by assuming equal weight on all phylogenies using the CLANN program [64]. When the supertree does not support either the select-genes tree or the common-genes tree, the supertree topology is additionally as supplementary information at [25]. Please note that reconstructed supertrees themselves do not have branch length information. When needed, branch length information was estimated from the selected genes by forcing a supertree topology.

Average branch length was used as an indicator for the degree of the divergence in that group. The method to identify members of a gene family has been described in [8]. This study focuses on the presence/absence pattern of each gene family rather than individual gene; thus, varied number of genes (e.g. duplicated genes) in a gene family within the group of organisms would not be taken into consideration in the analysis. Non-annotated genes were recovered from the whole genome DNA sequences using a TBLASTN search [65] with annotated genes as query sequences, and predicted ORFs that are present in only one genome but do not have homologues detected in any other complete genomes by BLAST were removed from further analysis. In addition, genes encoding proteins that are less than 100 amino acids in length were removed from further analysis in this study, since a similarity search using BLAST has less power to detect homologues in short sequences [65].

Informational genes in each genome were identified by applying the COG classification (Clusters of Orthologous Groups of proteins) [66]. All available protein sequences with functional annotation from bacterial genomes were downloaded from the COGs database [67]. There are 24,797 genes from 50 complete bacterial genomes involved in information storage and processing according to the COG classification (categories J, A, K, L, and B in COGs). A reciprocal BLASTP search was conducted to identify the homologues of informational proteins in the studied genomes. Significant hits were required to have expect values less than 10^-20 ^and match over 85% of the length of the query protein (10^-20 ^+ 85%). Different cutoffs (10^-10 ^+ 70%, 10^-05 ^+ 50%) were also examined to avoid the ambiguity of one cutoff threshold (Additional file 10). Genes that have significant hits with any informational genes were identified as informational genes. Rate variation for gene insertions/deletions was estimated after informational genes were excluded, and for comparisons, the same number of the most conserved genes were excluded and the same number of randomly chosen genes were removed. Rate variation for insertions/deletions of informational genes was estimated in the same manner.

## Authors contributions

WH and GBG designed the study. WH carried out all analyses. WH and GBG wrote the manuscript.

## Supplementary Material

Additional file 1 Different ins/del rates between informational genes and non-informational genes. A, estimation was based on the select-genes trees; B, estimation was based on the common-genes trees. Only constant rates with no rate variation are shown, and the *y *= *x *line is also shown. Click here for file

Additional file 2 Insertion/deletion rates of non-informative genes in different phylogenetic groups estimated with rate variation. Estimation was based on the select-genes trees. All the informational genes were excluded from the estimation. Click here for file

Additional file 3 Insertion/deletion rates between informational genes and noninformational genes in COG classification. Estimation was based on the select-genes trees. Click here for file

Additional file 4 Information on phylogeny construction using different methods. Click here for file

Additional file 5 Boxplot of tree length of the select-genes tree and the common-genes tree from each group. Group names are shown in the first three letters (except MYB for Mycobacterium, MYP for Mycoplasma. For each group, tree length of the select-genes tree is on the left, and that of the common-genes tree is on the right. Click here for file

Additional file 6 Alpha values based on different phylogenies. Estimation are based on possible alternative phylogenies for the common genes, which are sorted from best supported to lest supported. Click here for file

Additional file 7 Alpha values based on different phylogenies. Estimation are based on possible alternative phylogenies for the selected genes, which are sorted from best supported to lest supported. Click here for file

Additional file 8 The deletion of ribosomal proteins in Streptococcus mutans UA159 (GenBank accession: AE014133). Click here for file

Additional file 9 Small *α *change after excluding informational genes compared with excluding the most conserved genes. A, Estimation was based on the select-genes trees; B, Estimation was based on the common-genes trees. Each bar represents a group and all groups were sorted according to their ratios. The ratios are obtained from Table 5. Click here for file

Additional file 10 *α *value after informational genes were removed using different cutoffs on e-value and match length in identifying informative genes. Estimation was based on the select-genes trees. Maximum likelihood estimation was conducted by only using the best supported phylogeny of each group to reduce computational burden. Click here for file

Additional file 11 Simulation methods and results. Click here for file
