# Uncovering rate variation of lateral gene transfer during bacterial genome evolution

**DOI:** 10.1186/1471-2164-9-556

**Published:** 2008-11-25

**Authors:** Weilong Hao, G Brian Golding

**Affiliations:** 1 Department of Biology, McMaster University, Hamilton, Ontario, Canada, L8S 4K1

On page 11 of our publication [1], the correction for missing data in a discrete Γ distribution was conducted as

(1) $$Q_{+}=\prod_{i=1}^{N}L_{+}^{i}=\prod_{i=1}^{N}\sum_{j=1}^{M}p_{j}\frac{L^{i}(\mu_{j})}{1-L_{-}^{i}(\mu_{j})}.$$

This correction, equation (1), is only correct when the category probabilities *p*_*j *_have no effect on observability. Because the category probabilities have an effect on observability, the appropriate correction is

(2) $$Q_{+}=\prod_{i=1}^{N}L_{+}^{i}=\prod_{i=1}^{N}\frac{\sum_{j=1}^{M}p_{j}L^{i}(\mu_{j})}{1-\sum_{j=1}^{M}p_{j}L_{-}^{i}(\mu_{j})}.$$

Here *N *is the total number of gene families and *M *is the number of rate categories. Analyses based on equation (2) as opposed to equation (1) revealed remarkably similar results and all conclusions made based on the old method remain qualitatively unchanged. The additional analyses and the altered tables are provided in Additional File 1 and also available at . We thank Dr. Matt Spencer for pointing out the difference in averaging methods and thank Drs. David Bryant, Ofir Cohen, Miklós Csűrös, Tal Pupko, and Ed Susko for communication of ideas.

## Supplementary Material

Additional file 1 Click here for file
