# Supplementary material for: Uncovering rate variation of lateral gene transfer during bacterial genome evolution
Source: BMC Genomics. 2008 May 20;9:235. doi: 10.1186/1471-2164-9-235 (PMC2426709; doi:10.1186/1471-2164-9-235)
Supplement: Additional file 1 — Different ins/del rates between informational genes and non-informational genes. A, estimation was based on the select-genes trees; B, estimation was based on the common-genes trees. Only constant rates with no rate variation are shown, and the y = x line is also shown. [file 1471-2164-9-235-S1.pdf]

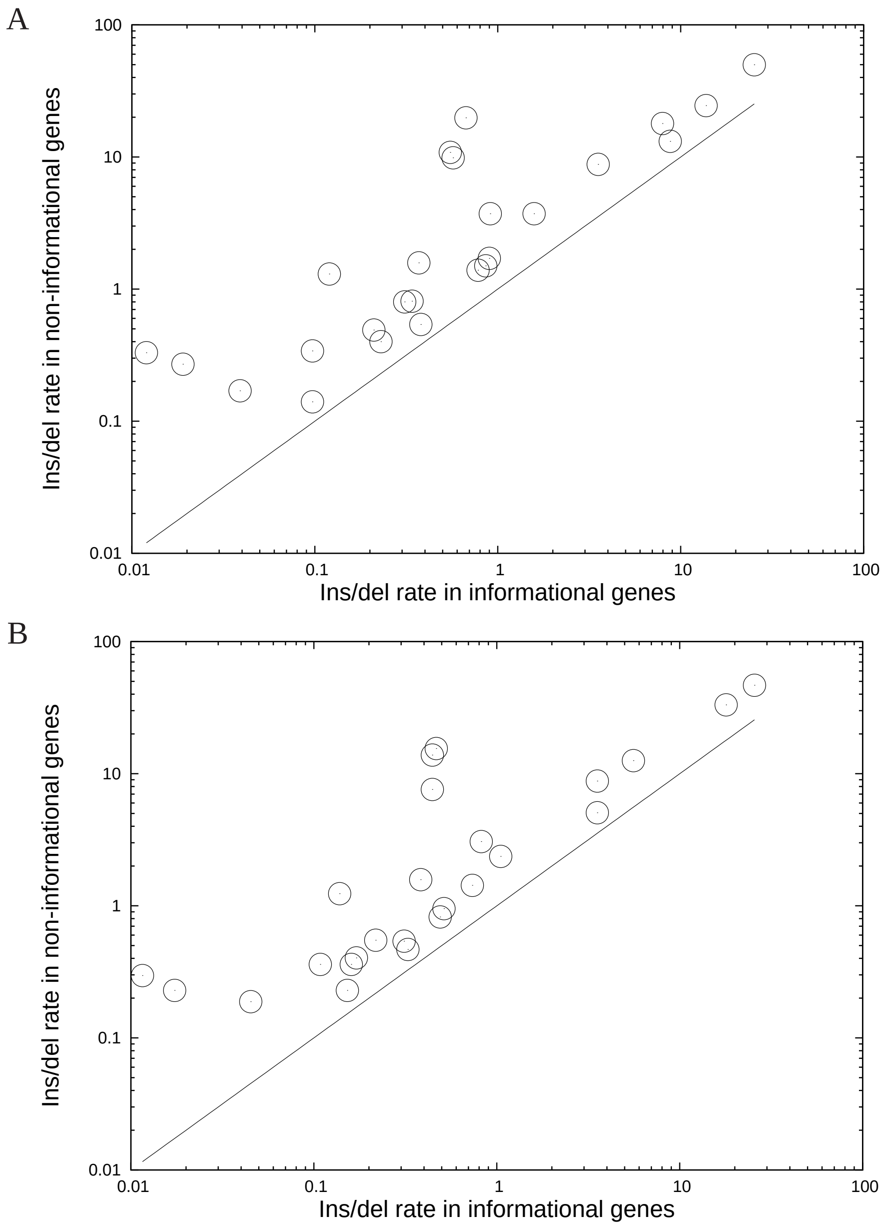

Figure S.1: Different ins/del rates between informational genes and non-informational genes. A, estimation was based on the select-genes trees; B, estimation was based on the common-genes trees. Only constant rates with no rate variation are shown, and the $y = x$ line is also shown.
