# Supplementary material for: Uncovering rate variation of lateral gene transfer during bacterial genome evolution
Source: BMC Genomics. 2008 May 20;9:235. doi: 10.1186/1471-2164-9-235 (PMC2426709; doi:10.1186/1471-2164-9-235)
Supplement: Additional file 2 — Insertion/deletion rates of non-informative genes in different phylogenetic groups estimated with rate variation. Estimation was based on the select-genes trees. All the informational genes were excluded from the estimation. [file 1471-2164-9-235-S2.pdf]

Table S.1: Insertion/deletion rates of non-informative genes in different phylogenetic groups estimated with rate variation. Estimation was based on the select-genes trees. All the informational genes were excluded from the estimation.

Group	Taxa	Genome Size	Length	Rate Variation			Constant Rate		ΔLnL
				α value	MLE	LnL	MLE	LnL	
Bacillus	13	4.9	0.126886	0.39	6.41	-37382	0.54	-37602	220*
Brucella	4	2.1	0.000775	0.057	95.94	-1070	24.51	-1108	38*
Burkholderia	7	3.8	0.028971	0.37	25.58	-16005	3.72	-16401	396*
Candidatus	4	1.3	0.608223	∞	0.40	-3992	0.40	-3992	0
Chlamydophila	7	1.2	0.081640	0.54	0.41	-1336	0.27	-1354	18*
Clostridium	5	3.3	0.123806	0.59	12.79	-8557	10.85	-8671	114*
Corynebacterium	5	3.0	0.172207	1.01	1.94	-7623	0.80	-7670	47*
Ehrlichia	5	1.5	0.059080	∞	0.32	-789	0.33	-789	0
Escherichia	7	5.0	0.002852	0.26	48.45	-9565	13.16	-10018	453*
Helicobacter	5	1.7	0.232504	0.59	1.76	-3634	1.30	-3734	100*
Lactobacillus	6	2.2	0.250368	∞	0.49	-8374	0.49	-8374	0
Mycobacterium	6	4.2	0.070373	0.49	18.13	-13338	1.72	-13367	29*
Mycoplasma	12	0.9	0.441286	∞	0.094	-7234	0.094	-7234	0
Prochlorococcus	5	1.9	0.356990	3.05	0.18	-4171	0.17	-4175	4*
Pseudomonas	8	6.3	0.057175	2.16	1.65	-25143	1.39	-25244	101*
Rhodopseudomonas	4	5.3	0.060450	0.70	2.60	-8638	1.5	-8706	68*
Rickettsia	5	1.3	0.055962	0.44	3.70	-2862	1.58	-2962	100*
Salmonella	5	4.8	0.002930	0.44	13.30	-4851	8.79	-4915	64*
Shigella	6	4.6	0.003188	0.39	43.74	-8884	17.91	-9145	261*
Staphylococcus	13	2.8	0.034391	0.097	285.34	-10129	19.76	-12626	2497*
Streptococcus	19	2.0	0.041459	0.37	12.57	-21785	9.85	-23595	1810*
Synechococcus	5	2.6	0.364362	∞	0.34	-6884	0.34	-6881	0
Vibrio	5	3.2	0.104170	0.23	10.94	-7867	0.81	-8111	244*
Xanthomonas	6	5.1	0.024790	0.37	16.33	-8878	3.54	-9153	275*
Yersinia	6	4.6	0.000263	0.070	271.98	-2954	49.81	-3282	328*

*Significant improvement
