# Supplementary material for: Uncovering rate variation of lateral gene transfer during bacterial genome evolution
Source: BMC Genomics. 2008 May 20;9:235. doi: 10.1186/1471-2164-9-235 (PMC2426709; doi:10.1186/1471-2164-9-235)
Supplement: Additional file 3 — Insertion/deletion rates between informational genes and noninformational genes in COG classification. Estimation was based on the select-genes trees. [file 1471-2164-9-235-S3.pdf]

Table S.2: Insertion/deletion rates between informational genes and noninformational genes in COG classification. Estimation was based on the select-genes trees.

Group	Informational Genes						Non-informational Genes[a]					
	Rate Variation			Constant Rate		ΔLnL	Rate Variation			Constant Rate		ΔLnL
	α value	MLE	LnL	MLE	LnL		α value	MLE	LnL	MLE	LnL	
Bacillus	0.25	2.88	-2757	0.38	-2917	160*	0.56	1.17	-12717	0.47	-13034	317*
Brucella	0.083	73.94	-139	13.62	-142	3*	0.025	91.11	-628	16.17	-649	21*
Burkholderia	0.23	9.92	-1207	1.58	-1259	52*	0.47	4.12	-5319	1.65	-5413	94*
Candidatus	∞	0.23	-629	0.23	-629	0	∞	0.38	-2713	0.38	-2713	0
Chlamydophila	0.022	0.031	-47	0.021	-47	0	0.069	0.082	-219	0.045	-220	1
Clostridium	0.19	8.25	-911	0.55	-983	72*	0.47	7.33	-4401	1.30	-4596	195*
Corynebacterium	0.17	4.06	-839	0.31	-862	23*	0.68	1.20	-3444	0.49	-3488	44*
Ehrlichia	∞	0.015	-15	0.015	-15	0	∞	0.012	-37	0.012	-37	0
Escherichia	0.14	51.45	-1109	9.21	-1206	97*	0.13	21.91	-3655	5.57	-3876	221*
Helicobacter	0.056	4.46	-269	0.11	-322	53*	0.39	0.83	-1579	0.28	-1643	64*
Lactobacillus	0.39	0.42	-842	0.21	-873	31*	1.91	0.46	-3814	0.37	-3828	14*
Mycobacterium	8.84	5.18	-1309	0.90	-1310	1	4.10	4.08	-5287	1.14	-5292	5*
Mycoplasma	1.94	0.08	-1016	0.07	-1021	5*	4.96	0.11	-2424	0.10	-2427	3*
Prochlorococcus	1.00	0.042	-193	0.039	-194	1	0.66	0.11	-1329	0.084	-1340	11*
Pseudomonas	0.43	1.34	-1855	0.75	-1927	72*	0.73	1.28	-10159	0.88	-10386	227*
Rhodopseudomonas	0.14	7.40	-679	0.86	-707	28*	0.328	1.94	-3379	0.86	-3437	58*
Rickettsia	0.14	0.94	-269	0.37	-282	13*	0.22	1.38	-1001	0.55	-1038	37*
Salmonella	0.12	6.33	-395	3.72	-408	13*	0.23	5.47	-1851	3.72	-1876	25*
Shigella	0.18	22.30	-827	7.96	-869	42*	0.27	22.94	-4055	9.83	-4208	153*
Staphylococcus	0.040	206.07	-1230	0.67	-1687	457*	0.10	8.03	-3157	0.47	-3602	445*
Streptococcus	0.15	7.82	-1909	0.58	-2213	304*	0.38	3.40	-6446	0.79	-6725	279*
Synechococcus	∞	0.097	-376	0.097	-376	0	∞	0.19	-2433	0.19	-2433	0
Vibrio	0.11	5.01	-713	0.34	-722	9*	0.70	0.79	-3809	0.49	-3833	24*
Xanthomonas	0.080	19.84	-453	0.95	-491	38*	0.16	8.49	-2196	1.23	-2280	84*
Yersinia	0.025	171.72	-269	25.63	-309	40*	0.031	152.33	-1278	23.63	-1404	126*

[a]Genes not in COG classification were excluded

*Significant improvement
