# Supplementary material for: Uncovering rate variation of lateral gene transfer during bacterial genome evolution
Source: BMC Genomics. 2008 May 20;9:235. doi: 10.1186/1471-2164-9-235 (PMC2426709; doi:10.1186/1471-2164-9-235)
Supplement: Additional file 4 — Information on phylogeny construction using different methods. [file 1471-2164-9-235-S4.pdf]

Table S.3: Information on phylogeny construction using different methods.

Group	Number of genes (characters) used in analysis					Supported By Supertree
	Select		Common		Supertree[a]	
	Gene	Characters	Gene	Characters	Genes	
Bacillus	7	9231	386	396175	3407	Common
Brucella	10	12576	591	669428	591	Common
Burkholderia	11	16755	634	679984	2126	Common
Candidatus	9	11553	134	167530	-	
Chlamydophila	16	24405	637	738291	-	
Clostridium	11	15216	309	338459	1141	Common
Corynebacterium	16	24546	578	724565	-	
Ehrlichia	15	21306	517	577079	-	
Escherichia	14	20556	1700	1673111	2980	Select
Helicobacter	14	20613	584	650282	-	
Lactobacillus	13	19326	339	376764	923	Neither
Mycobacterium	13	20751	607	679689	-	
Mycoplasma	9	12780	79	108760	433	Neither
Prochlorococcus	14	21993	617	706435	-	
Pseudomonas	16	23553	579	660179	3102	Neither
Rhodopseudomonas	13	19371	1140	1237551	-	
Rickettsia	15	20736	266	326738	-	
Salmonella	16	23118	1580	1570712	-	
Shigella	13	19155	1476	1459347	2608	Common
Staphylococcus	13	17559	565	596478	1902	Select
Streptococcus	12	17961	400	431646	1690	Neither
Synechococcus	15	21090	630	735413	-	
Vibrio	13	19386	971	1036551	1780	Common
Xanthomonas	5	5235	321	377134	-	
Yersinia	14	23118	1260	1308476	2752	Neither

[a]If the select-genes tree is topologically identical to the common-genes tree, a supertree was not constructed.
