# Supplementary material for: Uncovering rate variation of lateral gene transfer during bacterial genome evolution
Source: BMC Genomics. 2008 May 20;9:235. doi: 10.1186/1471-2164-9-235 (PMC2426709; doi:10.1186/1471-2164-9-235)
Supplement: Additional file 5 — Boxplot of tree length of the select-genes tree and the common-genes tree from each group. Group names are shown in the first three letters (except MYB for Mycobacterium, MYP for Mycoplasma. For each group, tree length of the select-genes tree is on the left, and that of the common-genes tree is on the right. [file 1471-2164-9-235-S5.pdf]

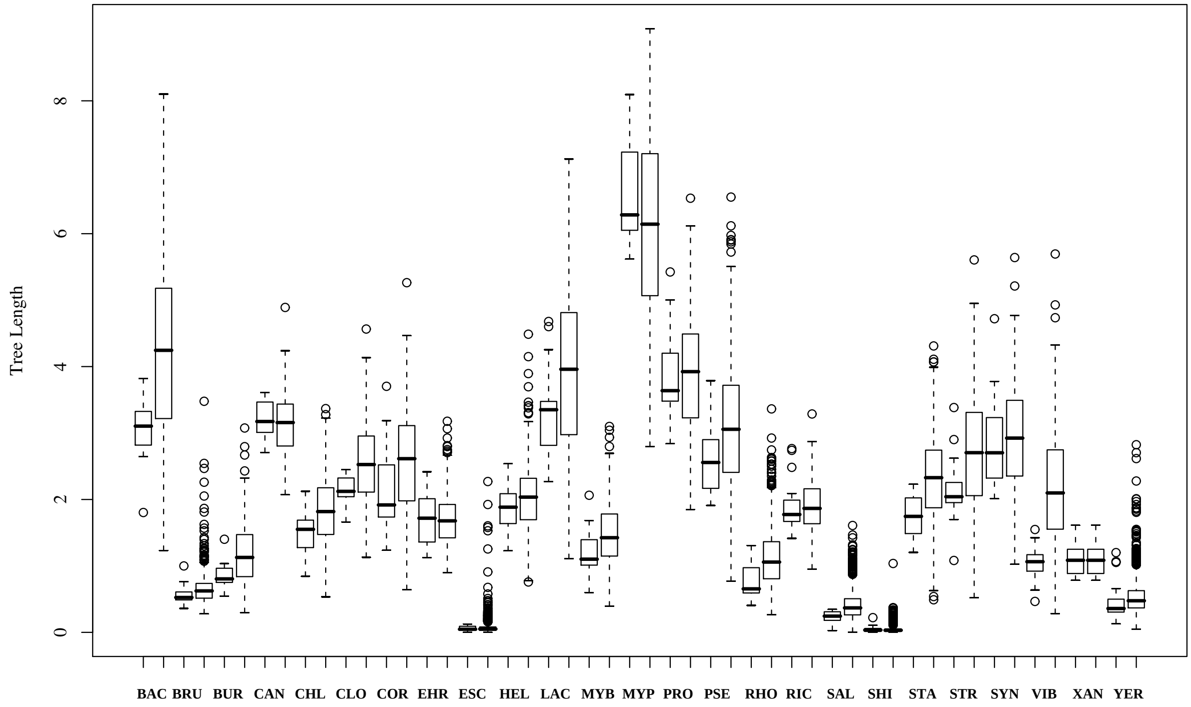

Figure S.2: Boxplot of tree length of the select-genes tree and the common-genes tree from each group. Group names are shown in the first three letters (except MYB for *Mycobacterium*, MYP for *Mycoplasma*. For each group, tree length of the select-genes tree is on the left, and that of the common-genes tree is on the right.)
