# Supplementary material for: Uncovering rate variation of lateral gene transfer during bacterial genome evolution
Source: BMC Genomics. 2008 May 20;9:235. doi: 10.1186/1471-2164-9-235 (PMC2426709; doi:10.1186/1471-2164-9-235)
Supplement: Additional file 6 — Alpha values based on different phylogenies. Estimation are based on possible alternative phylogenies for the common genes, which are sorted from best supported to lest supported. [file 1471-2164-9-235-S6.pdf]

Table S.4: Alpha values based on different phylogenies. Estimation are based on possible alternative phylogenies for the common genes, which are sorted from 1 to 10 as from best supported to lest supported.

Group	α in overall genes										α in Non-informational genes									
	1	2	3	4	5	6	7	8	9	10	1	2	3	4	5	6	7	8	9	10
Bacillus	0.39	0.35	0.39	-	-	-	-	-	-	-	0.39	0.41	0.39	-	-	-	-	-	-	-
Brucella	0.035	0.022	0.022	-	-	-	-	-	-	-	0.037	0.022	0.022	-	-	-	-	-	-	-
Burkholderia	0.18	-	-	-	-	-	-	-	-	-	0.19	-	-	-	-	-	-	-	-	-
Candidatus	∞	-	-	-	-	-	-	-	-	-	∞	-	-	-	-	-	-	-	-	-
Chlamydophila	0.39	-	-	-	-	-	-	-	-	-	0.52	-	-	-	-	-	-	-	-	-
Clostridium	0.52	-	-	-	-	-	-	-	-	-	0.63	-	-	-	-	-	-	-	-	-
Corynebacterium	1.19	-	-	-	-	-	-	-	-	-	1.70	-	-	-	-	-	-	-	-	-
Ehrlichia	∞	-	-	-	-	-	-	-	-	-	∞	-	-	-	-	-	-	-	-	-
Escherichia	0.31	0.31	-	-	-	-	-	-	-	-	0.33	0.33	-	-	-	-	-	-	-	-
Helicobacter	0.52	-	-	-	-	-	-	-	-	-	0.70	-	-	-	-	-	-	-	-	-
Lactobacillus	∞	-	-	-	-	-	-	-	-	-	∞	-	-	-	-	-	-	-	-	-
Mycobacterium	0.23	0.23	0.24	-	-	-	-	-	-	-	0.26	0.26	0.24	-	-	-	-	-	-	-
Mycoplasma	∞	∞	∞	∞	∞	∞	∞	∞	-	-	∞	∞	∞	∞	∞	∞	∞	∞	-	-
Prochlorococcus	2.56	-	-	-	-	-	-	-	-	-	3.44	-	-	-	-	-	-	-	-	-
Pseudomonas	1.91	1.91	1.70	1.91	1.91	-	-	-	-	-	2.28	2.15	2.15	2.28	2.28	-	-	-	-	-
Rhodopseudomonas	0.52	-	-	-	-	-	-	-	-	-	0.59	-	-	-	-	-	-	-	-	-
Rickettsia	0.35	-	-	-	-	-	-	-	-	-	0.41	-	-	-	-	-	-	-	-	-
Salmonella	0.080	0.085	0.085	0.085	0.080	-	-	-	-	-	0.095	0.095	0.095	0.101	0.095	-	-	-	-	-
Shigella	0.17	-	-	-	-	-	-	-	-	-	0.18	-	-	-	-	-	-	-	-	-
Staphylococcus	0.085	0.090	0.085	0.085	0.080	-	-	-	-	-	0.090	0.090	0.090	0.090	0.085	-	-	-	-	-
Streptococcus	0.29	0.29	0.29	-	-	-	-	-	-	-	0.33	0.31	0.33	-	-	-	-	-	-	-
Synechococcus	∞	-	-	-	-	-	-	-	-	-	∞	-	-	-	-	-	-	-	-	-
Vibrio	0.17	-	-	-	-	-	-	-	-	-	0.19	-	-	-	-	-	-	-	-	-
Xanthomonas	0.59	-	-	-	-	-	-	-	-	-	0.70	-	-	-	-	-	-	-	-	-
Yersinia	0.060	0.056	0.056	0.056	0.056	0.060	0.056	0.056	0.056	0.056	0.067	0.060	0.060	0.063	0.060	0.063	0.067	0.063	0.063	0.060
