# Supplementary figures and images for: Uncovering rate variation of lateral gene transfer during bacterial genome evolution
Source: BMC Genomics. 2008 May 20;9:235. doi: 10.1186/1471-2164-9-235 (PMC2426709; doi:10.1186/1471-2164-9-235)

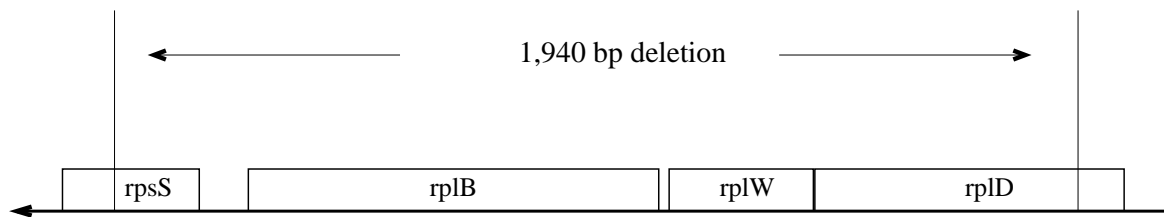

Figure S.3: The deletion of ribosomal proteins in *Streptococcus mutans* UA159 (GenBank accession: AE014133).

Supplement: Additional file 8 — The deletion of ribosomal proteins in Streptococcus mutans UA159 (GenBank accession: AE014133). [file 1471-2164-9-235-S8.pdf]
