# Supplementary material for: Uncovering rate variation of lateral gene transfer during bacterial genome evolution
Source: BMC Genomics. 2008 May 20;9:235. doi: 10.1186/1471-2164-9-235 (PMC2426709; doi:10.1186/1471-2164-9-235)
Supplement: Additional file 9 — Small α change after excluding informational genes compared with excluding the most conserved genes. A, Estimation was based on the select-genes trees; B, Estimation was based on the common-genes trees. Each bar represents a group and all groups were sorted according to their ratios. The ratios are obtained from Table 5. [file 1471-2164-9-235-S9.pdf]

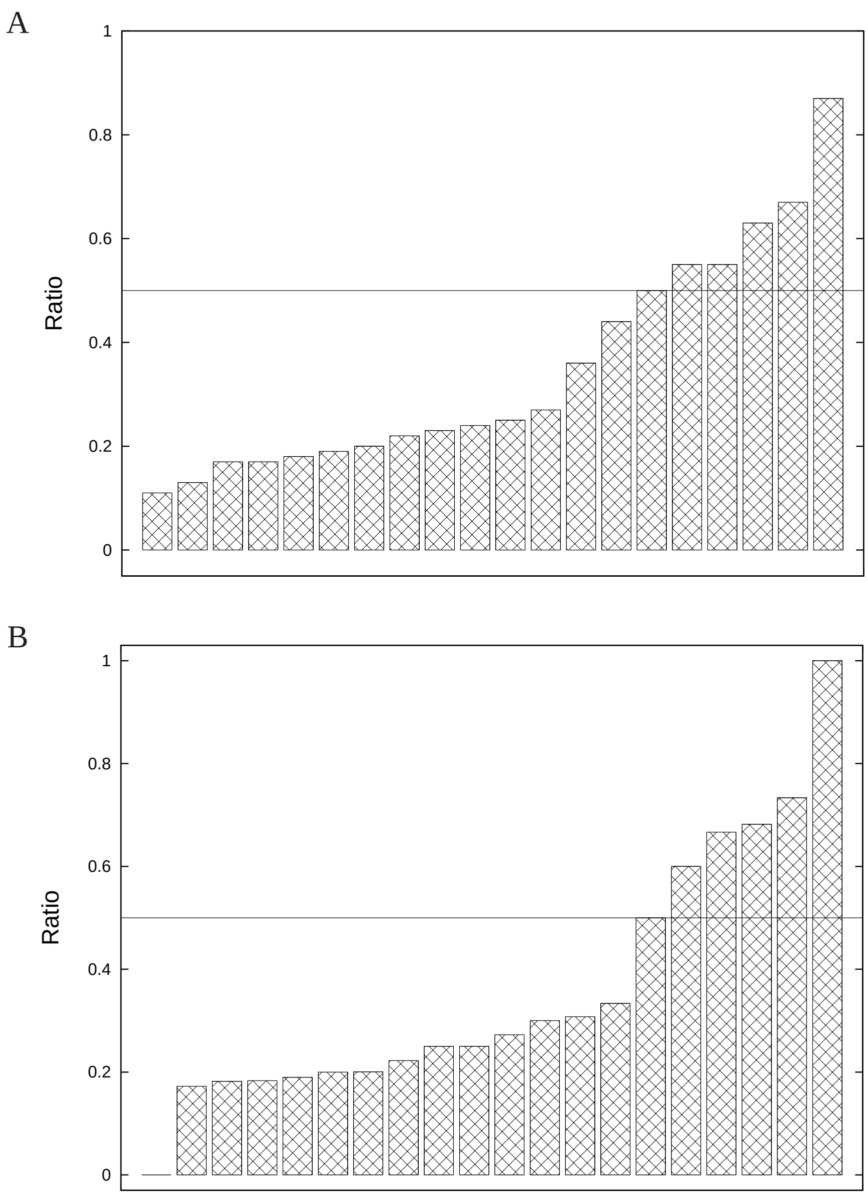

Figure S.4: Small α change after excluding informational genes compared with excluding the most conserved genes. A, Estimation was based on the select-genes trees; B, Estimation was based on the common-genes trees. Each bar represents a group and all groups were sorted according to their ratios. The ratios are from Table 4.
