# Supplementary material for: Uncovering rate variation of lateral gene transfer during bacterial genome evolution
Source: BMC Genomics. 2008 May 20;9:235. doi: 10.1186/1471-2164-9-235 (PMC2426709; doi:10.1186/1471-2164-9-235)
Supplement: Additional file 10 — α value after informational genes were removed using different cutoffs on e-value and match length in identifying informative genes. Estimation was based on the select-genes trees. Maximum likelihood estimation was conducted by only using the best supported phylogeny of each group to reduce computational burden. [file 1471-2164-9-235-S10.pdf]

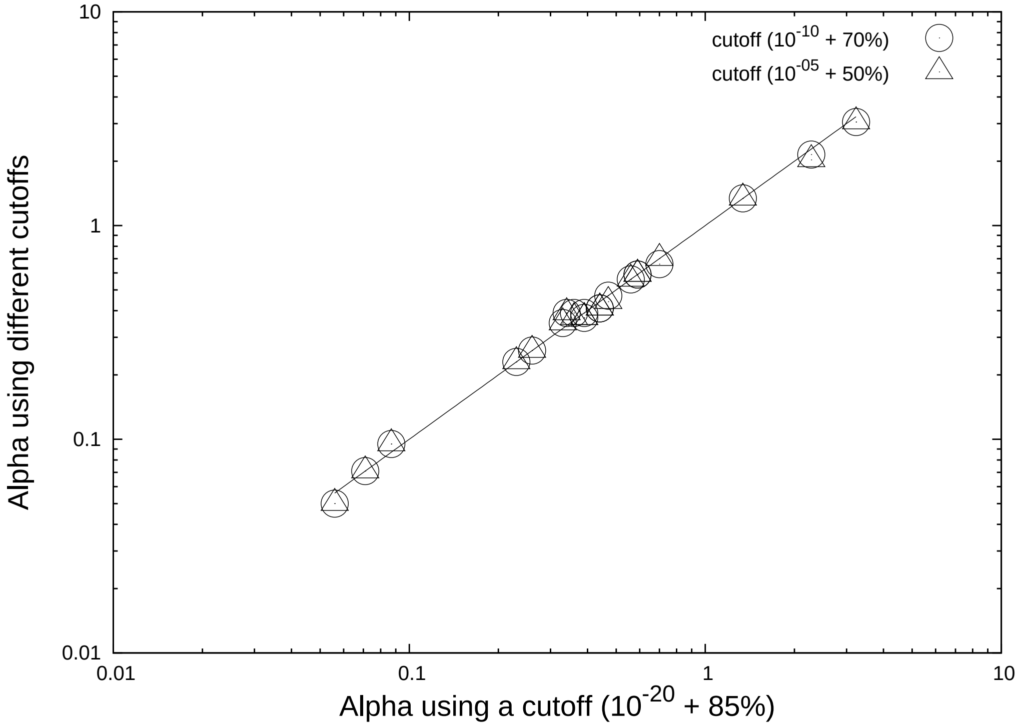

Figure S.5: α value after informational genes were removed using different cutoffs on e-value and match length in identifying informative genes. Estimation was based on the select-genes trees. Maximum likelihood estimation was conducted by only using the best supported phylogeny of each group to reduce computational burden.
