# Supplementary material for: Uncovering rate variation of lateral gene transfer during bacterial genome evolution
Source: BMC Genomics. 2008 May 20;9:235. doi: 10.1186/1471-2164-9-235 (PMC2426709; doi:10.1186/1471-2164-9-235)
Supplement: Additional file 11 — Simulation methods and results. [file 1471-2164-9-235-S11.pdf]

Supplementary Information

Simulation

In this study, phyletic patterns were used to estimate insertion/deletion rates by assuming changes of presence or absence in gene placeholders as in [1]. Some other methods, such as birth and death models, have also been used in modeling bacterial genome evolution [2–4]. Here, we took the advantage of varied methods, a simulation was conducted by assuming a simplistic birth-death process in a stochastic fashion (Figure S.6) and the simulated data were used for maximum likelihood estimation. In brief, a certain number of novel genes were introduced into a genome, and on average the same amount of genes were deleted from the genome based on their probability to be deleted (relative deletibility) during a given time period. All inserted genes were assigned to have a relative deletibility as 1. Different deletibilities were assigned for ancient genes in the simulations. It is noteworthy that the genes with small deletibility are less likely to be deleted compared with the inserted ones, but they are not free of deletion. Simulated results following a topology as in Figure S.7 were obtained to estimate the ins/del rates using a maximum likelihood method.

The simulated data were used to estimate the rates of ins/del by incorporating rate variation in a single rate model as done in the 25 groups. Figure S.8A shows that there was no detectable rate variation when the deletibility in ancient genes is equal to 1, and the level of rate variation increased when the deletibility in ancient genes became smaller. When the deletibility in ancient genes was small, lower levels of rate variation on insertions/deletions were observed (Figure S.8B). There was also a decrease at the level of rate variation along with the number of insertions/deletions increasing. In the simulated genomes, when the number of insertions/deletions was increased, the genomes would have more inserted genes and less ancient genes, even though the observed insertions/deletions tend to be greatly underestimated (data not shown). In the simulation, the difference between the small deletibility in ancient genes and the large deletibility (e.g. 1) in inserted genes contributed to rate variation. However, the inferred rate variation parameter α values from the simulated data are still dramatically higher than those from the genuine genomic data (Tables 2 and 3). This suggests that there is a large amount of rate variation in ancient genes and possibly inserted genes as well.

References

[1] Hao W, Golding GB: **The fate of laterally transferred genes: Life in the fast lane to adaptation or death.** *Genome Res* 2006, **16**:636–643.

[2] Berg OG, Kurland CG: **Evolution of microbial genomes: sequence acquisition and loss**. *Mol Biol Evol*. 2002, **19**:2265–2276.

[3] Gu X, Zhang H: **Genome phylogenetic analysis based on extended gene contents**. *Mol Biol Evol* 2004, **21**:1401–1408.

[4] Novozhilov AS, Karev GP, Koonin EV: **Mathematical modeling of evolution of horizontally transferred genes**. *Mol Biol Evol* 2005, **22**:1721–1732.

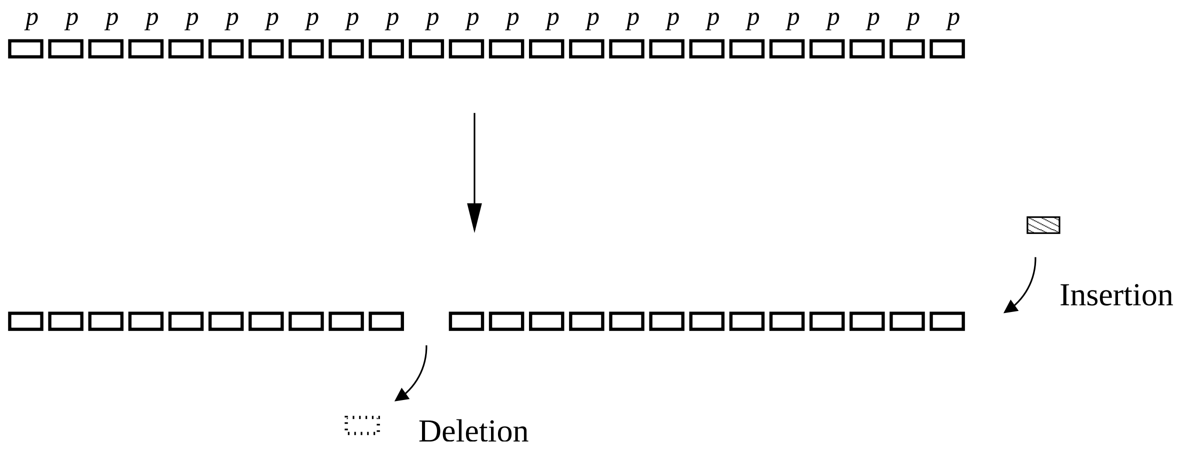

Figure S.6: Demonstration of a simple simulation process. Each gene has a probability to be deleted (deletibility). The relative deletibility of inserted genes is 1.

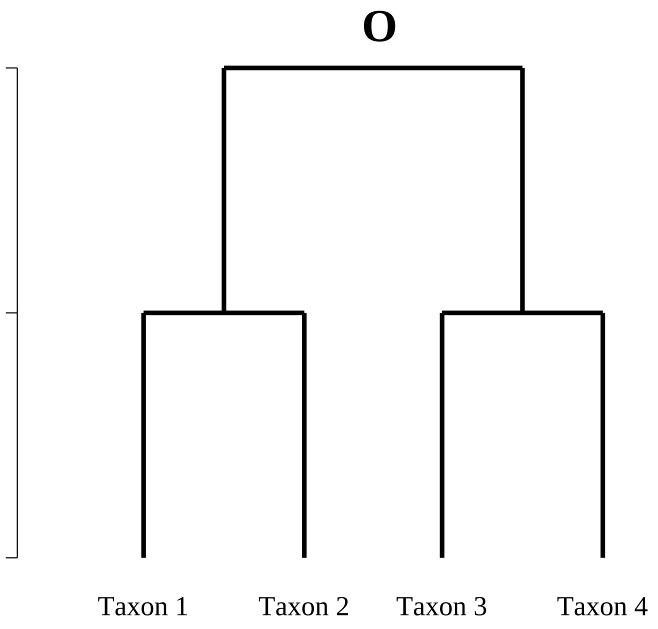

Figure S.7: A simple phylogeny used in the simulation. The length of external branches was assigned equal to internal branch lengths in all simulations.

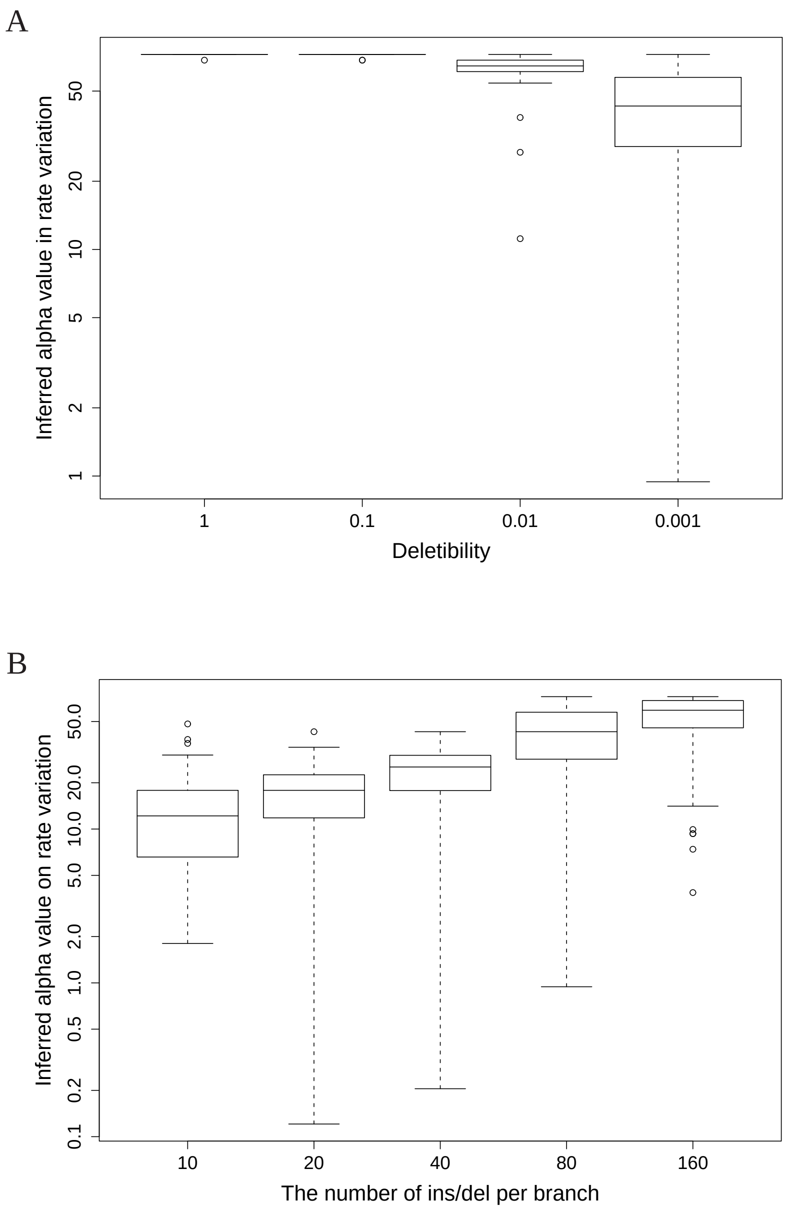

Figure S.8: Box plot of inferred rate variation parameter α values from the maximum likelihood estimation on simulated data. The maximum likelihood estimation was based on a single rate model by incorporating a Γ distribution. A, 80 insertions/deletions per branch with varied deletibilities for ancient genes; B, deletibility equal to 0.001 with varied insertions/deletions per branch.
