# Supplementary material for: Uncovering rate variation of lateral gene transfer during bacterial genome evolution
Source: BMC Genomics. 2008 Nov 25;9:556. doi: 10.1186/1471-2164-9-556 (PMC2628915; doi:10.1186/1471-2164-9-556)
Supplement: Additional file 1 [file 1471-2164-9-556-S1.pdf]

Correction: Uncovering rate variation of lateral gene transfer during bacterial genome evolution

Weilong Hao[1] and G. Brian Golding*[1]

[1]Department of Biology, McMaster University, Hamilton, Ontario, Canada L8S 4K1

Email: WH: haow@indiana.edu; GBG: golding@mcmaster.ca;

*Corresponding author

Corrigendum

The method in reference [1] used to correct for missing data (since genes absent in all of the taxa are unobservable) was

$$L_+ = \frac{L}{1 - L_-}.$$

In reference [2], a discrete Γ distribution was incorporated into the correction as

$$Q_+ = \prod_{i=1}^{N} L_+^i = \prod_{i=1}^{N} \sum_{j=1}^{M} p_j \frac{L^i(\mu_j)}{1 - L_-^i(\mu_j)}. \quad (1)$$

Here N is the total number of gene families, M is the number of rate categories. The right correction should be made as

$$Q_+ = \prod_{i=1}^{N} L_+^i = \prod_{i=1}^{N} \frac{\sum_{j=1}^{M} p_j L^i(\mu_j)}{1 - \sum_{j=1}^{M} p_j L_-^i(\mu_j)}. \quad (2)$$

Here, we test whether the method in equation (2) alters the results in [2].

The rate categories of a discrete Γ distribution in the new method were generated following [3] using the R package [4].

Analyses reveal that the method in eq.(2) does not qualitatively alter the results in [2]. Table SS.1 shows that the new method generated a slightly smaller α in each group, but the maximum likelihood values are remarkably similar with the ones reported in [2]. The association between α value and average branch length still holds as shown in Fig. SS.1. Furthermore, removing the informational genes has a more similar effect as random removal than removing the most conserved genes Table SS.2. In conclusion, the method results in slightly different α values and MLEs, but the conclusions made in [2] are robust to this more accurate correction method.

Acknowledgements

The authors thank Matt Spencer for pointing out the difference of averaging methods used for missing data correction and thank David Bryant, Ofir Cohen, Miklós Csűrös, Tal Pupko, and Ed Susko for communication of ideas.

References

1. Felsenstein J: **Phylogenies from restriction sites: A maximum- likelihood approach**. *Evolution* 1992, **46**:159–173.
2. Hao W, Golding GB: **Uncovering rate variation of lateral gene transfer during bacterial genome evolution**. *BMC Genomics* 2008, **9**:235.
3. Yang Z: **Maximum likelihood phylogenetic estimation from DNA sequences with variable rates over sites: approximate methods**. *J Mol Evol* 1994, **39**:306–314.
4. R Development Core Team: *R: A Language and Environment for Statistical Computing.* R Foundation for Statistical Computing, Vienna, Austria 2007, [http://www.R-project.org]. [ISBN 3-900051-07-0].

Table SS.1: Insertion/deletion rates among different phylogenetic groups estimated in rate variation model. Estimation was based on the common-genes trees and two correction methods were compared.

Group	Rate Variation (Hao and Golding 2008)				Rate Variation (eq. 2)				Constant Rate	
	α value	MLE	LnL	ΔLnL	α value	MLE	LnL	ΔLnL	MLE	LnL
Bacillus	0.39	3.89	-40017	261*	0.29	3.46	-40146	132*	-40278	0.44
Brucella	0.035	382.06	-1264	123*	0.022	258.59	-1265	122*	-1387	44.50
Burkholderia	0.18	181.93	-17467	924*	0.12	145.45	-17525	866*	-18391	2.14
Candidatus	∞	0.49	-4722	0	∞	0.50	-4722	0	-4722	0.49
Chlamydophila	0.39	0.30	-1439	27*	0.29	0.23	-1438	28*	-1466	0.19
Clostridium	0.52	8.49	-9529	227*	0.44	7.33	-9528	228*	-9756	7.23
Corynebacterium	1.19	2.48	-8492	17*	1.06	0.72	-8492	17*	-8509	0.49
Ehrlichia	∞	0.24	-831	0	∞	0.25	-831	0	-831	0.24
Escherichia	0.31	13.43	-10654	352*	0.23	8.91	-10650	356*	-11006	4.82
Helicobacter	0.52	1.75	-3961	148*	0.44	1.44	-3964	145*	-4109	1.05
Lactobacillus	∞	0.33	-9241	0	∞	0.32	-9241	0	-9241	0.33
Mycobacterium	0.23	191.03	-14660	25*	0.18	158.75	-14671	47*	-14718	1.63
Mycoplasma	∞	0.22	-8214	0	∞	0.22	-8214	0	-8214	0.22
Prochlorococcus	2.56	0.19	-4413	6*	2.03	0.18	-4412	7*	-4419	0.17
Pseudomonas	1.91	1.06	-26994	136*	1.60	0.96	-26995	135*	-27130	0.91
Rhodopseudomonas	0.52	1.75	-9352	107*	0.44	1.23	-9353	106*	-9459	0.77
Rickettsia	0.35	3.49	-3181	129*	0.26	2.25	-3174	136*	-3310	1.36
Salmonella	0.080	96.48	-5214	137*	0.042	84.19	-5218	133*	-5351	7.97
Shigella	0.17	131.93	-9784	543*	0.11	82.51	-9795	532*	-10327	11.39
Staphylococcus	0.085	279.41	-11390	3191*	0.060	225.65	-11441	3140*	-14581	14.76
Streptococcus	0.29	18.71	-24327	2226*	0.22	15.24	-24365	2188*	-26553	13.19
Synechococcus	∞	0.33	-7531	0	∞	0.34	-7531	0	-7531	0.33
Vibrio	0.17	10.02	-8642	260*	0.12	6.65	-8658	281*	-8939	0.37
Xanthomonas	0.59	6.33	-9385	156*	0.47	4.91	-9385	156*	-9541	2.73
Yersinia	0.060	221.14	-3172	295*	0.037	129.30	-3178	289*	-3467	31.15

*Significant improvement

Table SS.2: Different α values in a Γ distribution after excluding certain genes. Alpha values were estimated using the method given in eq. 2.

Group	Original	Genes removed			Difference[a]
		Random	Informational	Conserved	(Ratio)
Bacillus	0.291	0.291	3.439	12.489	0.26
Brucella	0.022	0.022	0.022	0.022	-
Burkholderia	0.121	0.121	0.136	0.162	0.37
Candidatus	∞	∞	∞	∞	-
Chlamydophila	0.291	0.291	0.414	0.414	1.00
Clostridium	0.439	0.414	0.525	1.060	0.17
Corynebacterium	1.060	0.943	1.509	2.714	0.32
Ehrlichia	∞	∞	∞	∞	-
Escherichia	0.230	0.230	0.244	0.308	0.18
Helicobacter	0.439	0.439	0.590	0.662	0.68
Lactobacillus	∞	∞	∞	∞	-
Mycobacterium	0.182	0.182	0.193	0.274	0.12
Mycoplasma	∞	∞	∞	∞	-
Prochlorococcus	2.028	2.028	2.876	5.503	0.24
Pseudomonas	1.600	1.600	2.028	3.439	0.23
Rhodopseudomonas	0.439	0.414	0.495	0.556	0.57
Rickettsia	0.259	0.244	0.309	0.391	0.44
Salmonella	0.042	0.042	0.047	0.050	0.62
Shigella	0.114	0.114	0.128	0.144	0.47
Staphylococcus	0.060	0.060	0.060	0.063	0.00
Streptococcus	0.217	0.217	0.244	0.328	0.24
Synechococcus	∞	∞	∞	∞	-
Vibrio	0.121	0.120	0.136	0.162	0.38
Xanthomonas	0.465	0.495	0.556	0.625	0.47
Yersinia	0.037	0.039	0.042	0.044	0.60

[a]The difference was calculated from $\frac{\alpha_{\texttt{informational}} - \alpha_{\texttt{random}}}{\alpha_{\texttt{conserved}} - \alpha_{\texttt{random}}}$.

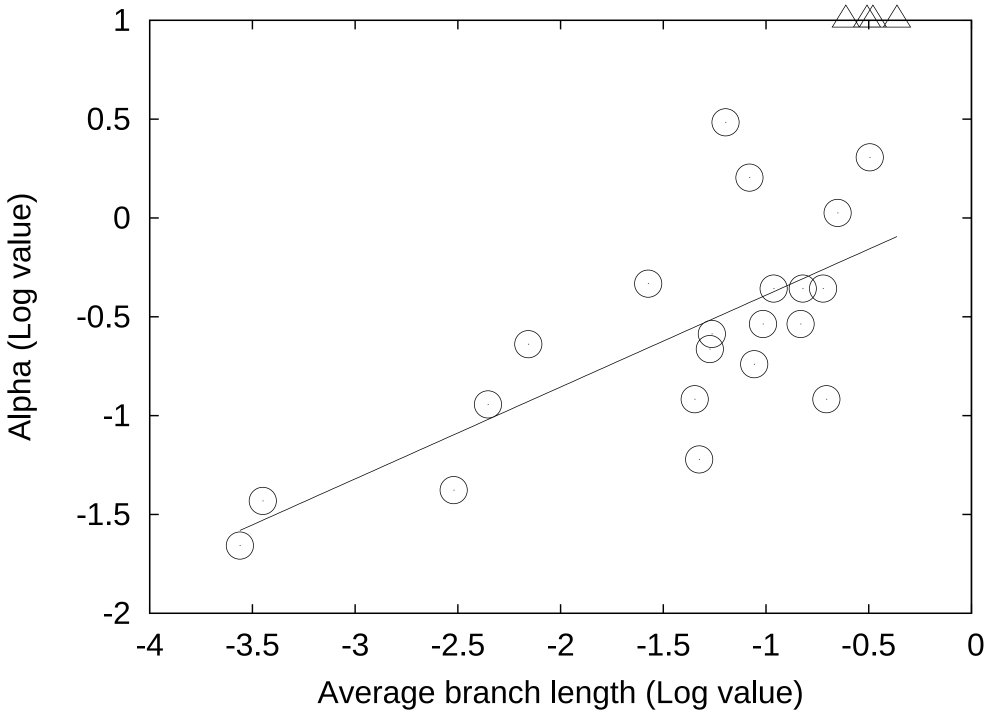

Figure SS.1: Average branch length and optimized α in a Γ distribution in each different group. The alpha values were estimated using the method in eq. 2. The five groups with an infinite α value are shown in open triangles. They were not used for estimating the regression line, $y = 0.465x + 0.074$ ($R^2 = 0.511$, $P \simeq 0.0003$).
